# Oseltamivir-resistant pandemic (H1N1)2009 in Yemen - case report

**DOI:** 10.1186/1743-422X-7-88

**Published:** 2010-05-08

**Authors:** Ahmed AK Thabet, Saeed H Al-Bahlooli, Abdulhakeem Al-Kohlani, Ahmed Shoja'a

**Affiliations:** 1 Community medicine department (communicable diseases epidemiology), faculty of medicine and health sciences, Thamar University, Sana'a road, Dhamar, Republic of Yemen; 2 General surgery department, faculty of medicine and health sciences, Thamar University, Sana'a road, Dhamar, Republic of Yemen; 3 Department of disease control and surveillance, Ministry of Public health and Population, Al-hasaba, Sana'a, Republic of Yemen

## Abstract

**Background:**

During the influenza season of 2007-08, oseltamivir-resistant influenza A (H1N1) viruses emerged in several countries in Europe, North America, and Asia. Despite substantial prevalence of oseltamivir-resistant viruses, few data are available on the clinical profile of subjects infected with these viruses. Objectives: to describe the first oseltamivir-resistant (H1N1) influenza virus pandemic 2009 from the Eastern Mediterranean Region including Yemen and to determine the evidence by clinical presentation of children infected with these oseltamivir - resistant viruses.

**Methodology:**

History, physical examination and laboratory investigations including Complete Blood Count, chest x-ray, blood cultures, CSF examination, LFTs, RFTs, blood for sugar, H1N1 test and oseltamivir resistance test.

**Results:**

Nasal swabs indicated positivity on both H1N1 test and the RNP gene (Human R Nase P gene that serves as internal positive control for Human RNA. Both clinical specimens presented the mutation S31N in the M2 gene associated with resistance to adamantanes and H274Y in NA gene associated with resistance to oseltamivir. This was the first diagnosed case of resistance to oseltamivir in Yemen and also it is the first reported case of oseltamivir resistance virus in the Eastern Mediterranean Region.

**Conclusion:**

The pattern of resistance found in the oseltamivir resistant isolate collected from Yemen is the same as has been reported elsewhere in other WHO regions. Clinical description and outcomes are not different from what is described elsewhere.

## Introduction

During the influenza season of 2007-08, oseltamivir-resistant influenza A (H1N1) viruses emerged in several countries in Europe, North America, and Asia. Despite substantial prevalence of oseltamivir-resistant viruses, few data are available on the clinical profile of subjects infected with these viruses [1]. In this paper we describe the first oseltamivir-resistant pandemic (H1N1) 2009 influenza virus from the Eastern Mediterranean Region including Yemen and determine the evidence of clinical presentation of children infected with these viruses regarding the occurrence of oseltamivir-resistance.

## Case report

3 years old female patient, known case of congenital heart disease with pulmonary hypertension and cerebral palsy was admitted to the hospital on 7^th ^October 2009 with high grade of fever, cough, sore throat, difficulty in breathing and body aches. Clinical examination: patient was semiconscious, disoriented and severe pale. Heart: Pan-systolic-murmur. Chest: bronchial breathing and crepitation on the right lung. Abdomen: soft, mild hepatomegaly. On the day of the hospitalization diagnosed as severe acute respiratory infection and admitted to the intensive care unit (ICU) under ventilator. Lab-Investigation was done (CBC, chest x-ray, blood culture, CSF examination, LFTs, RFTs, blood for sugar and blood culture). The results of lab-test were within normal range except lymphocytosis. It gives a suggestion that the infection could be viral and may due to H1N1.

On 9^th ^October 2009 nasal swab was taken for H1N1 test and introduce oseltamivir (Tamiflu) to the list of treatment. On the same day the result (RT-PCR) confirmed the diagnosis: H1N1 complicated severe acute respiratory infection; the patient spent more than 10 days in the ICU without clinical improvement. On review, repeat chest x-ray showed features of slight improvement. Second nasal swab was taken for H1N1 test. The result was still positive with high titration. The worsening picture suggested that the case could be resistant to oseltamivir. On 19^th ^October 2009 two throat swabs were taken and send to the EMRO for more confirmation and testing for resistance.

On 23^rd ^October 2009 test results from NAMRU-3 indicated that both specimens were positive for pandemic H1N1 and positive for the RNP gene (Human R Nase P gene that serves as internal positive control for Human RNA). Both clinical specimens presented the mutation S31N in the M2 gene associated with resistance to adamantanes. This mutation is present in all pandemic H1N1 viruses. Both clinical specimens contained the mutation H274Y in NA gene associated with resistance to oseltamivir. It was the first case of resistance to oseltamivir in Yemen and also it is the first case of oseltamivir resistance virus in the Eastern Mediterranean Region [2].

### Drug treatments

During hospitalization (from 7^th ^to 31 October 2009) patient was under several drugs including antibiotics such as (cephalosporin, makrolides, and clindamycine). Oseltamivir start on 9^th ^October and stopped on 19^th ^October 2009.

On 31 October 2009, we stopped all treatment except multivitamins and nutrition. The patient (1^st ^November 2009) clinically improved with no fever and difficulty in breathing

## Discussion

Pandemic influenza A (H1N1) was first identified in Yemen on 17th June 2009. By the end of February, the number of lab-confirmed H1N1 cases had reached 629 including 17th deaths associated with a case fatality rate 3%. Since the beginning of current influenza pandemic, a total of 40 cases of oseltamivir-resistant pandemic (H1N1) 2009 virus including this one from Yemen [3].

The dramatic rise of oseltamivir resistance in the H1N1 serotype in the 2007/2008 season^3^, and the fixing of H274Y in the 2008/2009 season have raised concerns regarding individuals at risk for seasonal influenza, as well as development of similar resistance in the H5N1 serotype3. Previously, oseltamivir resistance produced changes in H1N1 and H3N2 at multiple positions in treated patients. In contrast, the recently reported resistance involved patients who had not recently taken oseltamivir [4,5].

Moreover, the resistance was limited to the H1N1 which had acquired H274Y1, 2. H274Y jumped from clade2C (Hong Kong/2562/2006-like) to clade 1 (New Caledonia/20/1999-like) to clade 2B (Brisbane/59/2007-like) which included multiple introductions. Sub-clades that had acquired key changes on the neuraminidase and hemagglutinin genes expanded and fixed of H274Y on H1N1. These changes led to the spread of adamantane resistance on clade 2C outside of Asia, followed by the spread of oseltamivir resistance in 2007/2008 and the fixing of H274Y in 2008/2009.

The aggregation of key polymorphisms onto different genetic backgrounds supports a mechanism of homologous recombination between co-circulating influenza sub-clades, and provides a rationale for the prediction of vaccine targets and emergence of antiviral resistance [4,5].

The pattern of resistance found in the oseltamivir resistant isolate collected from Yemen is the same as has been reported elsewhere in other WHO Regions2. Prior studies of oseltamivir resistance in Japan5 were linked to sub-optimal dosing and resistance was found in both current influenza A subtypes, H1N1 and H3N2, and included, but was not limited to H274Y. Prior studies also supported a fitness penalty for the acquisition of H274Y, which predicted that the change would be limited to patients receiving oseltamivir [4,5].

Moreover, this isolate, and others collected over the summer in Hong Kong have a clade 2C HA and M2 which has H274Y in NA (both clade 2B and clade C) as well as S31N on M2, signaling additional exchanges of polymorphisms6 leading to the emergence of H1N1 which is resistant to oseltamivir and the adamantanes [4-6]. All these studies are going with the result of our case report.

The global experience with drug resistant pandemic influenza virus has shown that the risk of resistance is considered higher in patients who have prolonged illness (particularly those with severally compromised or suppressed immune system)and who have received antiviral treatment for an extended duration and still test positive for the virus^2^, this is an agreement with our case report; Moreover there is a strong need to monitor vigilantly for changes in transmissibility or pathogenicity of the pandemic (H1N1) 2009 influenza virus which may not be evident on a case-by case basis. Because of that any changes in the transmissibility and drug resistance of the virus will have pro-found implication on the clinical case management.

## Competing interests

The authors declare that they have no competing interests.

## Authors' contributions

All authors attest to having contributed substantially to conception and design and acquisition of data and drafting the article and revising it critically for important intellectual content. All authors give approval of the final version to be published.
